# Comparison of Corrosion Performance of Extruded and Forged WE43 Mg Alloy

**DOI:** 10.3390/ma15051622

**Published:** 2022-02-22

**Authors:** Guonan Liu, Jilei Xu, Baojing Feng, Jinhui Liu, Dongqing Qi, Wenzhan Huang, Peixu Yang, Shaojun Zhang

**Affiliations:** 1 Henan Province Industrial Technology Research Institute of Resources and Materials, Zhengzhou University, Zhengzhou 450001, China; dingshi95@126.com (G.L.); xjlclgc@163.com (J.X.); 13290904100@163.com (B.F.); yangpx@zzu.edu.cn (P.Y.); zhangs@zzu.edu.cn (S.Z.); 2 School of Materials Science and Engineering, Zhengzhou University, Zhengzhou 450001, China; 3 School of Chemistry and Chemical Engineering, Shandong University, Jinan 250100, China; 4 School of Materials Science and Engineering, Taiyuan University of Science and Technology, Taiyuan 030024, China; 2019063@tyust.edu.cn

**Keywords:** magnesium, rare earth elements, erosion, weight loss, pitting corrosion

## Abstract

Adjusting the microstructure through the deformation process is one of the ways to improve the properties of Mg alloys. Most studies have focused on the influence of the microstructure after deformation treatment on the mechanical properties of Mg alloys. In this paper, extruded and forged Mg-Gd-Y-Nd-Zr alloys were selected to investigate the corrosion performance of two deformed magnesium alloys immersed in 0.6 M NaCl solution using a hydrogen evolution test, a weight loss test, an immersion experiment, and an electrochemical test. The results showed that WE43 alloys undergoing different deformation treatments presented different microstructures, which led to different corrosion behaviors and corrosion resistance. The extruded WE43 alloy showed uniform corrosion, while the forged WE43 alloy suffered severe local galvanic corrosion. Meanwhile, the corrosion rate of the forged WE43 alloy was about four times faster than that of the extruded WE43 alloy.

## 1. Introduction

Magnesium (Mg) and Mg alloys have been attractive engineering materials for lightweight structural applications in the fields of aerospace, automotive, and electronics due to the combination of their high strength, low density, thermal conductivity, and electromagnetic shield ability [1,2]. At the same time, Mg alloys have been considered as a potential candidate for biodegradable cardiovascular and orthopedic implants due to their low density (1.7–2.0 g/cm^3^), relatively high stiffness, good biocompatibility, and similar elastic modulus (41–45 GPa) as the human bone [3,4,5,6]. In addition, in the field of energy, magnesium alloys are used as anode materials for high-power seawater activated batteries [7]. 

Magnesium alloys containing rare earth (RE) elements are considered as one of the most promising magnesium alloys due to improved mechanical properties, oxidation resistance, and creep resistance at both ambient and elevated temperatures, which can be attributed to the fact that RE elements may alter the alloy stacking fault energy (SFE) and raise the solute drag effects on the grain boundary or dislocation movements [8,9,10,11]. Moreover, the surface film containing RE has been reported to be more protective [12]. Among the developed Mg-Y, Mg-Gd, and Mg-Sc high-performance rare earth magnesium alloy systems, Mg-Gd-Y-Nd-Zr series alloys are the most successful. 

In general, commercially produced casting samples contain a large number of precipitates, which lead to a highly chemically heterogeneous microstructure and limit the mechanical property of magnesium alloys [13,14]. Therefore, a range of deformation processes, such as extrusion, forging, severe rolling, and other special processing techniques, have been developed to alter the microstructure [10,11,15,16,17,18,19,20]. Xia et al. [21] found that when Mg-6.85Gd-4.52Y-1.15Nd-0.55Zr alloy was multi-directionally forged, the dynamic precipitation was mainly concentrated at the grain boundary, which had a significant impact on grain refinement, and the amount of dynamic precipitation increased as the MDF passes increased. Liu et al. [18] obtained Mg-6Gd-3Y-2Nd-0.4Zr alloy with the ultimate tensile strength of 435 MPa through indirect hot extrusion and subsequent aging treatment, which can be attributed to the grain refinement effect of dynamic recrystallization and precipitation reinforcement. These studies indicate that the volume fraction, size, and distribution of the second phase alter during the deformation process.

The microstructure of magnesium alloys after deformation treatment changes, which also affects the corrosion resistance of the magnesium alloy. Liu et al. [22] studied the corrosion resistance of the cast and extruded EW75 rare earth magnesium alloy. The results showed that extruded EW75 had a lower volume fraction, smaller size, and more uniform distribution of second phase and exhibited better corrosion resistance than cast EW75. 

Previous studies have mainly focused on the microstructure and mechanical properties of Mg-Gd-Y-Nd-Zr alloys or the different corrosion resistance between the cast and deformed Mg alloys [18,23,24,25,26,27]. The relationship between the structure and corrosion resistance of magnesium alloys treated with different deformation processes has been seldom studied. In this work, extruded and forged WE43 magnesium alloys were selected to study the corrosion behavior of alloys treated with different deformation processes.

## 2. Experiments and Methods

### 2.1. Material Preparation

As-cast WE43 alloys were kindly supplied by Zhengzhou Light Metals Research Institute of Aluminum Corporation of China Limited (CHALCO, Zhengzhou, China). The nominal composition of the WE43 alloy was 1.55 wt % (0.26 at %) Gd, 3.79 wt % (1.11 at %) Y, 2.43 wt % (0.44 at %) Nd, and 0.5 wt % (0.14 at %) Zr and Mg balance, in accordance with ASTM B951-2011 [28]. Before the deformed processes, the ingots were solid solution treated at 525 °C for 20 h in an argon atmosphere and then rapidly quenched into water at 80 °C. After solution treatment, a part of the ingots was extruded with the extrusion ratio of 10:1 and extrusion rate of 0.4 mm/s at 400 °C. The other part of the ingots was forged in air by an industrial air pneumatic hammer machine (C41-750) with a load gravity of 400 kg. This impact forging process is similar to the multiple forging process (MFD) [21]. Before forging, the homogenized alloy blocks were placed in a resistance furnace and kept at a temperature of 400 °C for 1 h. Then, the samples were exposed to a small pass strain of ~0.05, and the average strain rate was around 50 s^−1^. The forging of one surface was counted as one pass, and the forging direction changed 90° after each pass. The entire forging process included 30 passes.

The experimental samples with dimensions of 10 × 10 × 10 mm^3^ and 30 × 30 × 10 mm^3^ were obtained from alloy ingots by wire cutting. The 10 × 10 × 10 mm^3^ sample was used for microstructure observation, immersion testing, and electrochemical test, while the 30 × 30 × 10 mm^3^ sample was used for hydrogen evolution test and weight loss test. Before the next test, SiC abrasive papers with grades from P120 to P3000 grit were selected to remove surface dirt and oxide film by wet grinding. The samples of extruded WE43 alloy were tested on the plane perpendicular to the extrusion direction (ED).

All tests were conducted at 25 °C ± 0.5 in 0.6 M NaCl solution prepared by dissolving NaCl in deionized water. The reagents (A.R. grade) were provided by Shanghai Aladdin Biochemical Technology Co. Ltd. In all experiments, at least three parallel experiments are performed to ensure experimental reliability.

### 2.2. Microstructural Characterization

The test surface of the specimens was polished with 0.5 μm diamond polishing paste at a speed of 1000 rpm through a polishing machine. Then, the polished specimens were rinsed with deionized water, degreased with absolute ethanol (95%), and dried in cold air.

After the grain boundaries of the polished sample were etched with 4 mL nitric acid + 96 mL ethanol solution, the optical metallographic (OM) photos were obtained by the ultra-depth-of-field three-dimensional microscope system VHX-2000E produced by Keyence Japan (Osaka, Japan).

The phase composition was analyzed by X-ray diffraction (XRD) using a DIFFRACTOMETER-6100 X-ray diffractometer (Shimadzu, Columbia, MD, USA) with Cu Kα radiation, and measurements were performed by continuous scanning of 2*θ* from 10° to 80° with a step size of 0.05° and a scanning speed of 4°/min. The phases were analyzed using MDI Jade software.

Element distribution and microstructure characteristics of the alloy surface were measured by a field-emission scanning electron microscope (SEM; American FEI Quanta 250 FEG, FEI, Hillsboro, OR, USA) with an accelerating voltage of 20 kV and a transmission electron microscope (TEM; Thermo-Fisher Talos F200X, Thermo Fisher Scientific, Waltham, MA, USA) equipped with Super-X energy-dispersive spectrometer (EDS) system. The high-angle annular dark-field scanning transmission image (HAADF-STEM) and the selected area electron diffraction (SAED) pattern were measured separately. The surface area fraction of the second phase on the alloy surface was counted using Image-Pro, and the evaluation materials were derived from the SEM-BSE images of two deformed alloys (three 600 × 510 um^2^ of each type)

### 2.3. Immersion Measurements

The specimens used for the immersion testing were treated with the same grinding and polishing process as the sample for the microstructure morphology. Then, specimens were exposed to 0.6 M NaCl solution at 25 °C ± 0.5 for 30 min, 2 h, 10 h, 1 day, 3 days, 5 days, and 7 days. After immersion, all samples underwent a process of cleaning with deionized water, degreasing with ethanol, and drying with cold airflow. Finally, the immersed samples were divided into three groups. The first group was sprayed with gold to study the surface characteristics of the corrosion product film; the second group was immersed in a chromic acid solution containing 180 g/L CrO_3_ for 5 min to remove corrosion products and study the corrosion characteristics; and the third group was first embedded in resin, then polished, and finally sprayed with gold to study the cross-sectional morphology of the surface film.

Hydrogen gas was collected through a combined collector composed of a funnel and a burette, and the combined collector was placed in a beaker containing 2 L of experimental solution. A 30 × 30 × 10 mm^3^ sample was hung in the funnel by a thread to avoid the occurrence of crevice corrosion, which is caused by contact with the beaker [22]. At the same time, a digital balance (JA3003) with an accuracy of 0.0001 g was used to obtain weight loss by weighing the mass of the samples before and after immersion. In addition, after immersing in 0.6 M NaCl solution at 25 °C ± 0.5 for 7 days, the sample was immersed in 180 g/L CrO_3_ solution for 10 min, washed with deionized water, degreased with ethanol, and weighed after being dried in cold air.

### 2.4. Electrochemical Measurements

All electrochemical tests were carried out at 25°C ± 0.5 in an electrochemical workstation (CHI660D) with a standard three-electrode configuration, and at least three parallel experiments were carried out to ensure repeatability. A sample embedded in epoxy resin with an exposure area of 1 cm^2^ was used as the working electrode, a platinum sheet was used as the counter electrode, and an R232 saturated calomel electrode (SCE) (+0.242 V vs. standard hydrogen electrode (SHE)) was used as the reference electrode. In addition, the three electrodes were immersed in 0.6 M NaCl electrolyte solution. Polarization curves were obtained at a scanning speed of 0.5 mV s^−1^, with the cathodic polarization curves starting at the OCP value and ending at a potential of −300 mV vs. OCP and the anodic polarization curves starting at the mV vs. OCP and terminating at the current density of 1 mA cm^−2^. Measurement of the polarization curve was started after the working electrode had stabilized for 30 min. The corrosion current density was obtained by extrapolation of the cathodic curve. The samples used for electrochemical impedance spectroscopy (EIS) were immersed in 0.6 M NaCl solution for 30 min. The EIS parameters were set to a disturbance voltage of 5 mV and an AC frequency ranging from 100 kHz to 0.01 Hz. The ZSimDemo 3.30 software was used to fit the EIS spectra. 

## 3. Results

### 3.1. XRD Analyses and Microstructural Characterization 

**Figure 1:**
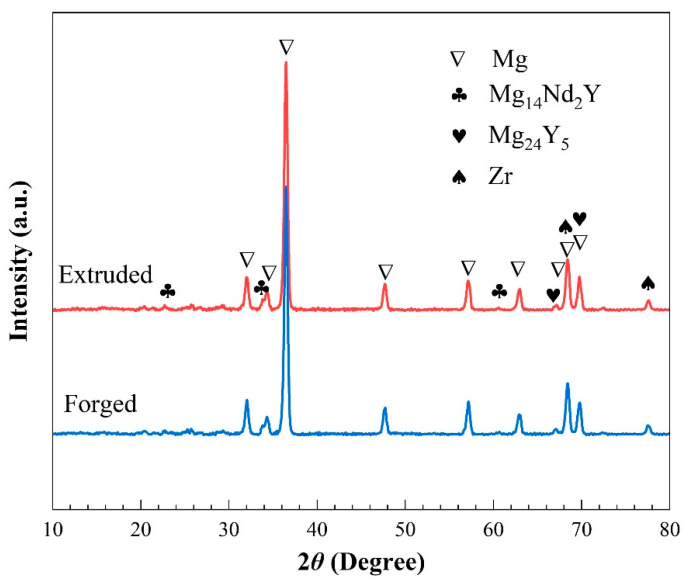
XRD patterns of experimental WE43 alloys.

The phase composition and microstructure of two deformed WE43 alloys were analyzed prior to the corrosion tests. Figure 1 shows the XRD diffraction patterns of the extruded and forged WE43 alloys. The diffraction peaks of the extruded WE43 alloys and the forged WE43 alloys had the same 2*θ* position, indicating that the alloys after the two deformation processes had the same phase composition. They were mainly composed of α-Mg, Mg_14_Nd_2_Y phases, and Mg_24_Y_5_ phases (PDF#31-0817), which is consistent with other studies [29].

**Figure 2:**
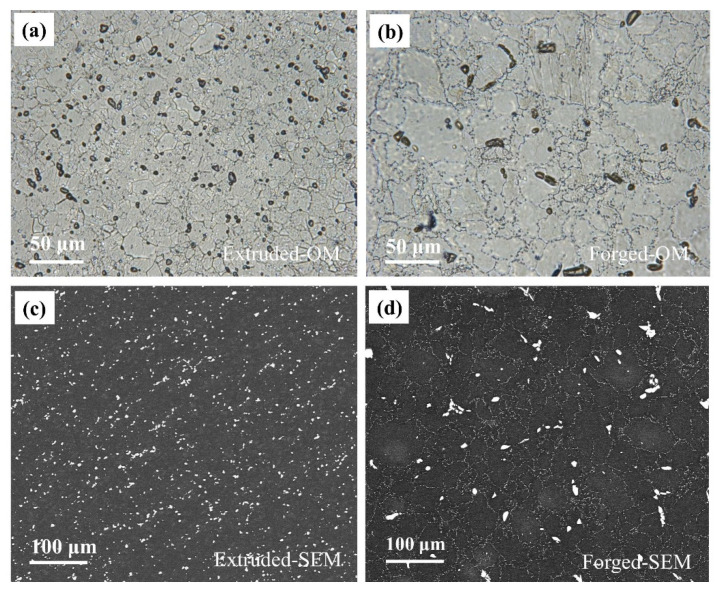
Optical microstructures and scanning electron microstructure of experimental WE43 alloys: (**a**,**c**) the extruded WE43 alloy; (**b**,**d**) the forged WE43 alloy.

Figure 2 shows the typical surface morphology of the extruded and forged surface-polished WE43 alloys. As can be seen from Figure 2a, the extruded WE43 alloy completed dynamic recrystallization, and the grains were uniform and fine equiaxed grains [23,30]. Meanwhile, a large number of second phases were similar in size and distributed along the grain boundaries. In the forged sample, the optical image (Figure 2b) showed uneven structure and existence of a small amount of coarse grains [8,19,30,31]. Moreover, two different morphologies of the second phase were observed, one with small particles distributed along the grain boundary and the other with a much larger strip. The grain boundary of the forged WE43 alloy was irregular bending. Figure 2c,d shows the position and proportion relationship between the second phase (bright parts) and the Mg matrix (dark regions). The second phase was dispersed and evenly distributed in the whole extruded sample structure (Figure 2c). However, the forged WE43 alloy showed a big difference. The small pearl-shaped second phase particles were distributed on the grain boundary, while the large-scale second phase particles were distributed randomly on the surface (Figure 2d). The statistical results of the second phase surface area fraction of the extruded and forged alloys were about 4.0% and 3.4%, respectively, and the grain size of the extruded and forged WE43 alloys were 18 and 40 μm, respectively.

**Figure 3:**
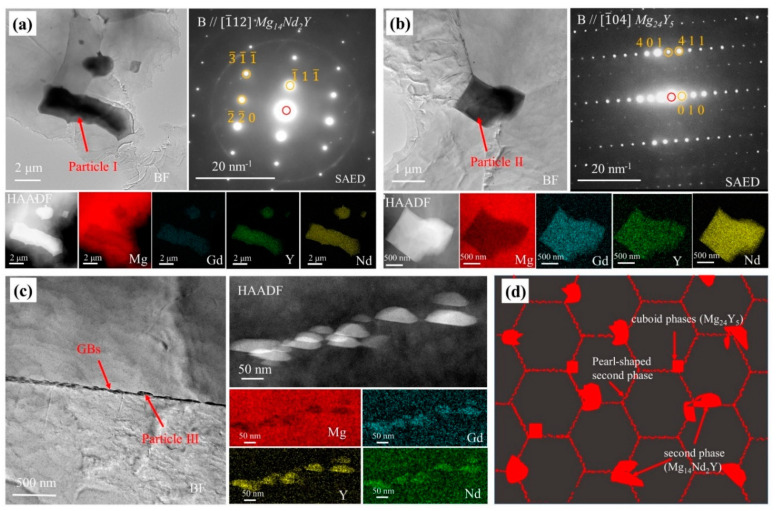
TEM bright-field images and corresponding SAED patterns, HAADF-STEM image and corresponding EDS elemental mappings, and schematic diagram of extruded WE43 alloy: (**a**) Particle I in extruded WE43 alloy; (**b**) Particle II in extruded WE43 alloy; (**c**) Particle III in extruded WE43 alloy; (**d**) schematic diagram of extruded WE43 alloy.

**Figure 4:**
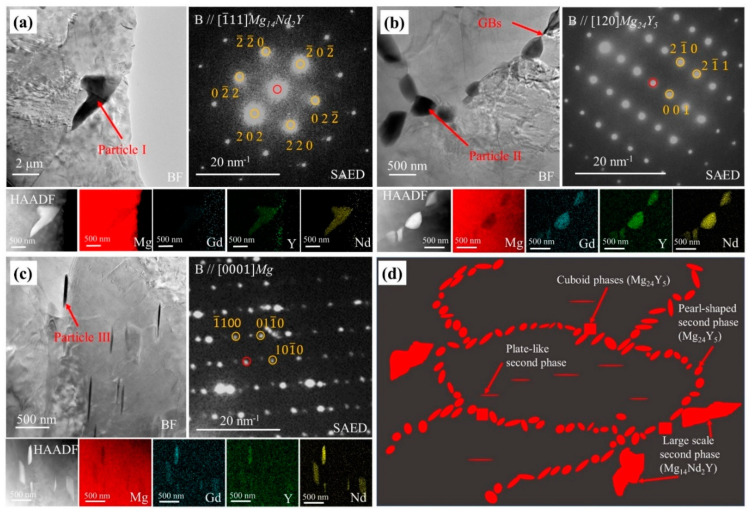
TEM bright-field images and corresponding SAED patterns, HAADF-STEM image and corresponding EDS elemental mappings, and schematic diagram of forged WE43 alloy: (**a**) Particle I in forged WE43 alloy; (**b**) Particle II in forged WE43 alloy; (**c**) Particle III in forged WE43 alloy; (**d**) schematic diagram of forged WE43 alloy.

For a clear observation of the microstructure, TEM analysis was performed. There were three morphological phases in the extruded specimen. Particle I in Figure 3a corresponded to the second phase in Figure 2c, whose SAED pattern was very close to that of Mg_14_Nd_2_Y [31]. Particle II in Figure 3b was confirmed as a cuboid phase, and its SAED pattern was consistent with the diffraction pattern of Mg_24_Y_5_. Meanwhile, clusters of particles (Figure 3c) with a size of about 70 nm were observed at grain boundaries. Four types of particles were observed in the forged specimen. Particle I in Figure 4a corresponded to the large-scale second phase in Figure 2d, and its diffraction pattern corresponded to the diffraction pattern of Mg_14_Nd_2_Y. Particle II in Figure 4b corresponded to the pearl-shaped second phase in Figure 2d, and its diffraction pattern was consistent with that of Mg_24_Y_5_. In addition, a small amount of plate-like particles similar to the aging treatment was observed inside the grains [32]. Similarly, there were cuboid phases in the forged specimen. According to the results of OM, SEM, and TEM, the structural schematic diagrams of the extruded and forged alloys are shown in Figure 3d and Figure 4d, respectively.

According to the EDS mappings in Figure 3 and Figure 4 and the chemical composition of alloys in Table 1, rare earth elements were enriched in these particles and the Nd content of these particles was higher than that of Y and Gd, which was different from the nominal composition of WE43 alloy. As Gd partially replaced Nd, the chemical composition of the particles in Figure 3a and Figure 4a can be denoted as Mg_14_(Nd, Gd)_2_Y. Similarly, Nd and Gd partially replaced Y, so the particles in Figure 3b and Figure 4b can be defined as Mg_24_(Nd, Gd, Y)_5_.

### 3.2. Immersion Test

#### 3.2.1. Corrosion Morphology Characterization

**Figure 5:**
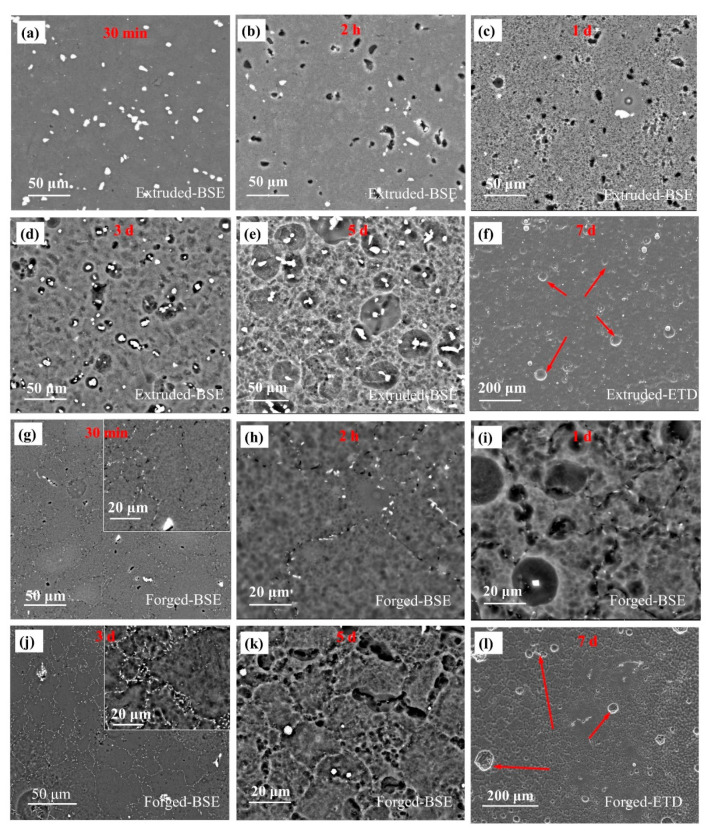
Corrosion morphologies of (**a**–**f**) the extruded WE43 alloy in 0.6 M NaCl solution for (**a**) 30 min, (**b**) 2 h, (**c**) 1 day, (**d**) 3 days, (**e**) 5 days, and (**f**) 7 days and (**g**–**l**) the forged WE43 alloy in 0.6 M NaCl solution for (**g**) 30 min, (**h**) 2 h, (**i**) 1 day, (**j**) 3 days, (**k**) 5 days, and (**l**) 7 days with corrosion product removed.

Figure 5 shows the surface morphology of the extruded and forged WE43 alloys after immersion in 0.6 M NaCl solution for a series of time and removal of corrosion products, respectively. As for the extruded WE43 alloy, the corrosion behavior can be divided into three steps. First, microgalvanic corrosion between the second phase and the Mg matrix occurred, and the falling off of the second phase could be observed after immersion for 2 h due to dissolution of the adjacent Mg matrix around the second phase (Figure 5a,b). Second, the dissolution of the Mg matrix dominated this step (Figure 5c), and plenty of micropits were present on the surface of the Mg matrix with immersion time of 1 day (Figure 5c). Third, with the dissolution of the Mg matrix, the surface of the Mg matrix turned smooth, the second phases underneath were exposed, and the microgalvanic corrosion was triggered again (Figure 5d). With a long immersion term, severe pitting corrosion around the second phase could be observed (Figure 5e,f).

Regarding the forged WE43 alloy, it mainly contained two kinds of second phase. The large-scale second phase caused more severe pitting corrosion than that in the extruded WE43 alloy after a long immersion time (see arrows in Figure 5f,l). On the other hand, the small pearl-shaped second phases followed the three steps mentioned for the extruded WE43 alloy. Figure 5g,h corresponds to the first step, Figure 5i corresponds to the second step, and Figure 5j corresponds to the third step. The difference is that the small pitting corrosion caused by the pearl-shaped second phase was distributed along the grain boundary, which seems to be intergranular corrosion (Figure 5l).

#### 3.2.2. Surface Film Characterization

**Figure 6:**
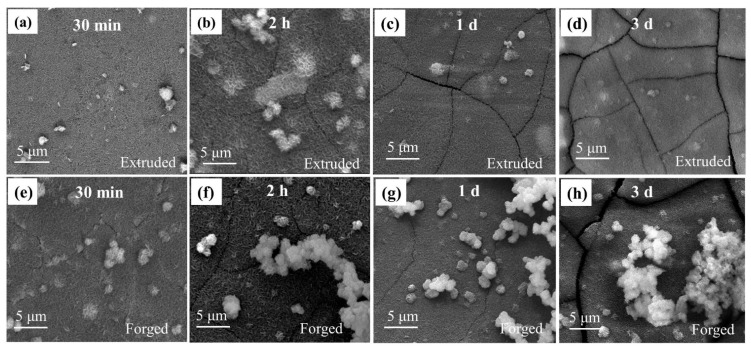
Surface morphologies of the (**a**–**d**) extruded and (**e**–**h**) forged WE43 alloys after immersion in 0.6 M NaCl solution for (**a**,**e**) 30 min, (**b**,**f**) 2 h, (**c**,**g**) 1 day, and (**d**,**h**) 3 days.

The micromorphologies of the surface film in the extruded and forged WE43 alloys after immersion in 0.6 M NaCl solution for different times are shown in Figure 6. The surface film composed of short fibrous particles was uniform and dense in the extruded WE43 alloy after immersion for 30 min (Figure 6a). Cracks occurred when the alloy was soaked for two hours (Figure 6b). With immersion time increasing, the cracks extended to a larger range (Figure 6c,d). The crack propagation can be attributed to the increase in film thickness. 

The corrosion crack propagation applied equally to the forged WE43 alloy, and the moment of crack emergence was earlier than the extruded WE43 alloy (Figure 6e). In addition, the appearance of the flocculent product indicated that the surface film was no longer dense and protective [33].

**Figure 7:**
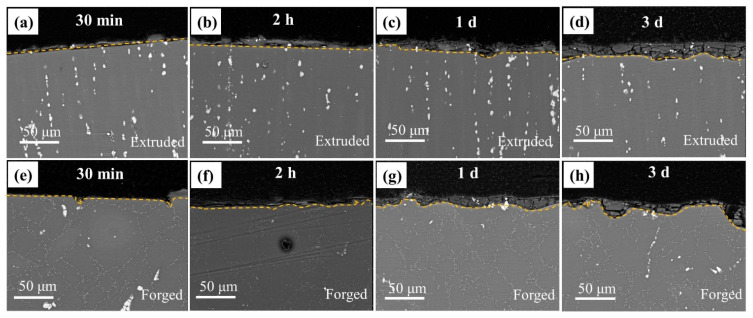
Cross-sectional morphologies of the (**a**–**d**) extruded and (**e**–**h**) forged WE43 alloys after immersion in 0.6 M NaCl solution for (**a**,**e**) 30 min, (**b**,**f**) 2 h, (**c**,**g**) 1 day, and (**d**,**h**) 3 days.

The cross-sectional morphology of the films presenting temporal evolution of corrosion layer formation is depicted in Figure 7. The microgalvanic corrosion of the extruded and forged WE43 alloys in 0.6 M NaCl solution was characterized by the dissolution of anodic magnesium and the undegraded second phase embedded in the corrosion product layer. During the period of exposure to 0.6 M NaCl solution, prolonging the exposure time would cause the corrosion layer to deepen. Additionally, the fluctuation of the corrosion depth of the extruded WE43 alloy was very small with no large corrosion pits (the yellow dotted line in Figure 7), while distinct corrosion pits were observed in the cross-sectional morphology of the forged WE43 alloy. Moreover, combined with the corrosion morphology of the corrosion product layer (Figure 6), the corrosion pits could be identified as the severe local corrosion between the large-scale phase and the Mg matrix.

#### 3.2.3. Gravimetric Results

**Figure 8:**
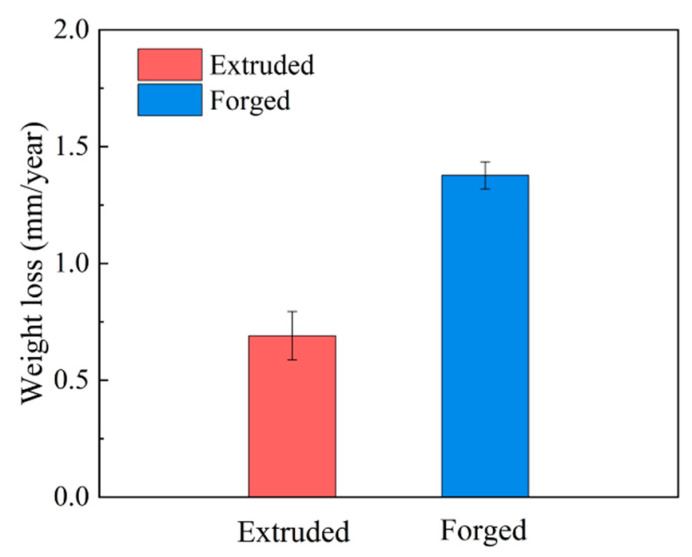
Weight loss rates of WE43 alloys after immersion in 0.6 M NaCl solution for 7 days.

To estimate corrosion resistance, the weight loss rates of two deformed WE43 alloys were obtained after immersion in 0.6 M NaCl solution for 7 days, and the results are summarized in Figure 8. The weight loss rates of the forged and extruded WE43 alloys immersed in 0.6 M NaCl solution for 7 days were 1.377 and 0.614 mm year^−1^, respectively. The weight loss rate of the forged WE43 alloy was approximately double that of the extruded WE43 alloy in NaCl solution during the immersion measurement period, implying that the forged WE43 alloy had suffered more severe corrosion damage. 

#### 3.2.4. Hydrogen Evolution Results

**Figure 9:**
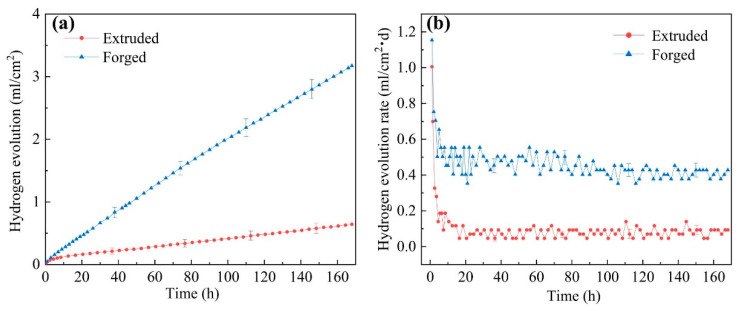
Average hydrogen evolution amount (**a**) and rate (**b**) of WE43 alloys in 0.6 M NaCl solution.

Figure 9 provides the hydrogen evolution volume variations and the hydrogen evolution rates of the alloys versus immersion time in 0.6 M NaCl solution. The forged WE43 sample exhibited a more serious corrosion behavior compared to the extruded WE43 alloy (Figure 9a). Simultaneously, the hydrogen evolution rate of WE43 alloys showed an identical tendency (Figure 9b). With prolongation of immersion time, the hydrogen evolution rate of WE43 alloys decreased and then basically stabilized, which was related to the formation of the surface film. 

### 3.3. Electrochemical Corrosion Behavior of WE43 Mg Alloy

#### 3.3.1. EIS Analysis

**Figure 10:**
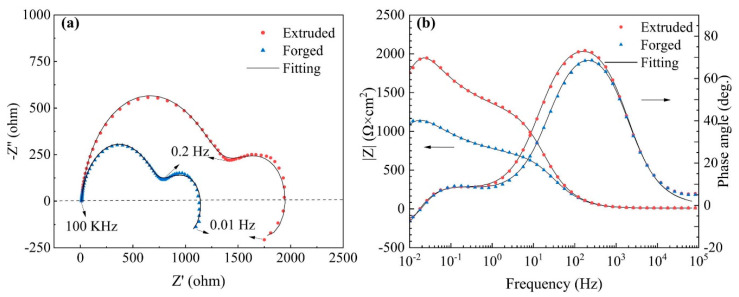
Electrochemical impedance spectrum (EIS) plots of the WE43 alloys: (**a**) Nyquist plots; (**b**) Bode plots.

The Nyquist and Bode spectra of the extruded and forged WE43 alloys after immersion in 0.6 M NaCl solution for 30 min are depicted in Figure 10. The Nyquist spectra of these alloys consisted of three semicircles: a high-frequency capacitance loop, a medium-frequency capacitance loop, and a low-frequency inductance loop. The diameter of the capacitance loop of extruded WE43 alloys was significantly larger than forged WE43 alloys, suggesting that the extruded WE43 alloy had better corrosion resistance than the forged WE43 alloy. Additionally, according to the bode plot in Figure 10b, the phase angle value and the impedance at the low frequency of the extruded WE43 alloy were larger than the forged WE43 alloy, implying that the extruded WE4 alloy had the best corrosion resistance.

**Figure 11:**
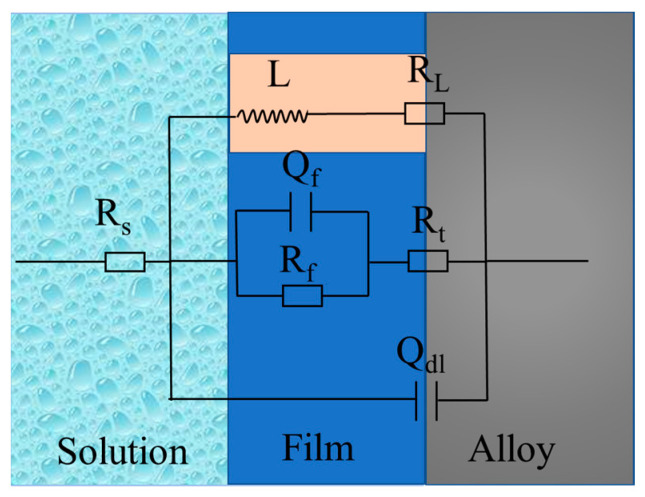
Equivalent circuits of EIS spectra.

In order to continuously clarify the corrosion mechanism of the alloys, the equivalent circuits and fitting data of the EIS spectra are provided in Figure 11 and Table 2. R_s_ represents the solution resistance. R_t_ and Q_dl_ describe the high-frequency capacitive loop. R_t_ is the charge transfer resistance, illustrating the transfer of electrons in the original substrate, and Q_dl_ stands for the electric double layer capacity. R_f_ and Q_f_ characterize the medium capacitance loop, originating from the diffusion through a porous solid film on the surface of the alloy. R_f_ represents the film resistance, and Q_f_ is the film capacitance. R_L_ and L describe the low-frequency inductive loop, which corresponds to corrosion nucleation of localized corrosion [34]. Localized corrosion can be observed in Figure 7e. According to the fitting results in Table 2, extruded WE43 alloys showed higher R_t_ and R_f_ values, indicating a slower dissolution rate and better film protection. The EIS results corresponded to those of weight loss measurements (Figure 8) and hydrogen evolution (Figure 9).

#### 3.3.2. Potential Dynamic Polarization Curves

**Figure 12:**
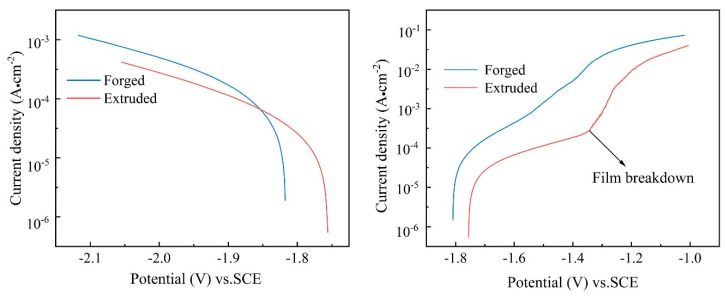
Comparison of polarization curves of extruded and forged WE43 alloys in 0.6 M NaCl solution: (**a**) cathodic side; (**b**) anodic side.

To estimate corrosion rates and interpret the corrosion mechanism, the cathodic and anodic polarization curves of the forged and extruded WE43 alloys were obtained after immersion in 0.6 M NaCl solution (Figure 12). The cathodic polarization curves of the forged and extruded WE43 alloys were very clearly similar, showing the characteristics of hydrogen evolution reaction. However, the anodic polarization curves, which are controlled by the anodic dissolution of Mg, displayed quite different results. An inflection that appeared to be a breakdown potential (E_bd_) was observed in the anode curve of the extruded sample, indicating that the surface film of the extruded sample could play a better protective effect than the forged WE43 alloy.

The corrosion potentials, cathodic Tafel slopes, and corrosion current densities measured by linear Tafel extrapolation are listed in Table 3. According to the fitting results, the E_corr_, I_corr_, and B_c_ of the forged WE43 alloy in NaCl solution were −1.82 V_SCE_, 140.3 μA cm^−2^, and 319.8 mV decade^−1^, while those of the extruded WE43 alloy were −1.75 V_SCE_, 33.1 μA cm^−2^, and 265.9 mV decade^−1^ (Table 3). Feng et al. [27] suggested that a higher Tafel slope could be ascribed to higher reaction resistance and lower theoretical efficiency of hydrogen evolution reaction (2H_2_O + 2e^−^→ H_2_ + 2OH^−^) at cathodic phase. As a consequence, the forged WE43 alloy in NaCl solution showed higher activity and faster corrosion rate than the extruded WE43 alloy in the initial corrosion stage, which corresponds to the hydrogen evolution results shown in Figure 9 and the Nyquist diagram in Figure 10.

## 4. Discussion

### 4.1. Different Microstructures of the Extruded and Forged WE43 Alloys

The WE43 alloy treated with the two deformation processes had the same phase composition but different microstructures, indicating that they were sensitive to the process condition. Combined with SEM and TEM analysis, it was observed that the difference between the precipitates of the extruded and forged samples was mainly manifested in the size. During the extrusion process, the high density of dislocations as well as large deformation energy promoted the precipitation of the second phase, and the particles of the second phase served as recrystallization nucleation points to promote dynamic recrystallization, resulting in the WE43 alloy exhibiting an equiaxed microstructure with complete dynamic recrystallization [25,27,29]. For the forged WE43 alloy, on the one hand, the rare earth element had a strong tendency to segregate at the grain boundary because its atomic size is larger than that of Mg to reduce the misfit energy. On the other hand, due to the severe plastic deformation during the forging process, the fast diffusion channel provided by the high dislocation density accelerated this segregation [32,35]. Moreover, due to the large misfit energy, the nonuniform segregation of rare earth elements during the forging process reduced the pinning effect of solute atoms, and the crystal grains showed abnormal grain growth.

### 4.2. Different Corrosion Behaviors of the Extruded and Forged WE43 Alloyws

Based on other experimental conclusions, the phase species, morphologies, volume fractions, and distributions regulate the microgalvanic effect in the corrosion and have an obvious influence on the performance of the film [12,36,37,38]. The results of weight loss, hydrogen evolution analysis, and electrochemistry indicated that the corrosion resistance of the extruded WE43 alloy was superior to the forged WE43 alloy.

Due to the dispersed distribution of the second phase, the extruded WE43 alloy exhibited a more electrochemically homogeneous microstructure, which reduced the intensity of galvanic corrosion, thereby transforming localized corrosion into uniform corrosion (Figure 5c). As the hydrogen evolution reaction stabilized (Figure 9), magnesium ions and hydroxide radicals were evenly distributed on the entire surface, forming uniform corrosion products on the surface of the extruded alloy, and the corrosion film suffered less lashing from hydrogen bubbles. The dense corrosion product film provided effective protection to the Mg matrix under the film, which was confirmed by the existence of the breakdown potential in the anodic polarization branch (Figure 12b).

After being immersed in 0.6 M NaCl solution, the forged alloy had obvious local corrosion pits and exhibited a nonuniform corrosion product film. The independent corrosion pits were due to the shedding of the large second phase. The shallow corrosion pits gradually extended to the grain interior and came into contact with each other (Figure 5i), which can be attributed to the unique continuous distribution and lower electrochemical activity of the pearl-shaped second phase. The characteristic flocculent products of the forged surface film originated from the combination of locally concentrated magnesium ions and hydroxide radicals, indicating that the alloys had undergone violent local corrosion (Figure 6). In addition, corrosion pits in the cross-sectional (Figure 7) and surface morphologies (Figure 5) indicated that destructive local corrosion had occurred and had penetrated into the interior. This phenomenon reveals that different cathode sizes might lead to different corrosion behaviors, and the large second phase has higher electrochemical activity than the pearl-shaped second phase.

The difference in microstructure between extruded and forged WE43 alloys was also reflected in the grain size and surface fraction of the second phase (Figure 2). The second phase fraction of extruded and forged WE43 alloys was 4.0% and 3.4%, respectively. According to the microgalvanic corrosion theory between the α-Mg matrix and the second phase [37,39], the increase in secondary phases typically accelerate the corrosion rate of Mg alloys, which is inconsistent with our experimental phenomenon. The grains of the extruded WE43 alloy was more uniform compared with the forged WE43 alloy (Figure 2), and the grain size of the extruded and forged WE43 alloys was 18 and 40 μm, respectively. Saikrishnaet al. [40] studied the effect of bimodal grain size distribution on corrosion behavior of AZ31 alloy and revealed that uniform grain size was beneficial to reduce corrosion rate in a high chloride ion environment. Ralston et al. [41] suggested that the corrosion rate is related to grain boundary length and that grain refinement increases the corrosion resistance. Thus, the small and uniform grain size made the extruded WE43 exhibit better corrosion resistance.

Texture engineering is an effective method to improve the mechanical properties of magnesium alloys [16]. This has been achieved through intelligent alloying and/or severe plastic deformation (SPD) programs for many years [13,17,42,43,44]. However, the addition of RE elements effectively weakens the deformation texture, thus improving the formability of the alloy. Therefore, when comparing the corrosion properties of the alloys treated with the two deformation processes, the texture was excluded as the influencing factor.

**Figure 13:**
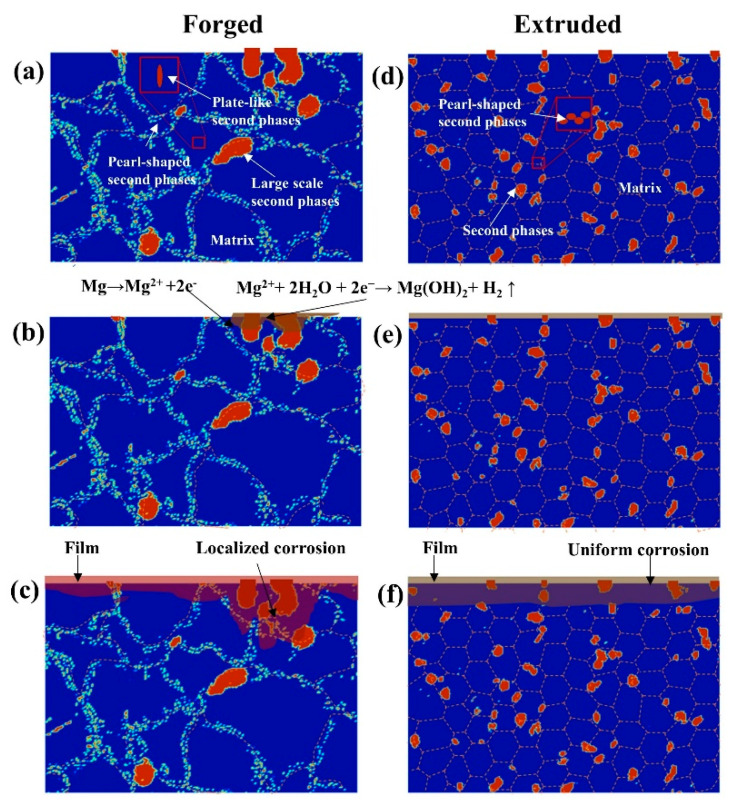
Schematic illustration of the development of surface film with increasing immersion time. Forged WE43 alloy: (**a**) polished, (**b**) immersion for a short time, and (**c**) immersion for a long time. Extruded WE43 alloy: (**d**) polished, (**e**) immersion for a short time, and (**f**) immersion for a long time.

Based on the above analysis, a sketch map was created to illustrate the corrosion process. Due to its high hardness, the second phase in the extruded and forged WE43 alloys protruded from the Mg matrix after polishing (Figure 13a,d). When the sample was immersed in the corrosion solution, corrosion reaction was triggered (Figure 13b,e). At this stage, the main corrosion process was microgalvanic corrosion between the second phase and the Mg matrix. Mg was dissolved through the reaction.

Mg → Mg^2+^ + 2e^−^ (1)

In the meantime, with the dissolution of magnesium, the film began to form [45].

Mg^2+^ + 2H_2_O + 2e^−^ → Mg(OH)_2_ + H_2_ ↑ (2)

Henceforth, the whole sample surface was gradually overspread by the corrosion product film (Figure 13c,f). For the extruded WE43 alloy, the distribution of the second phase was uniform, so there was no strong local corrosion. For the forged WE43 alloy, the large second phase acted as a strong cathode and formed a microgalvanic effect with the adjacent Mg matrix. The Mg matrix around the large-scale cathode phase was dissolved to form a concave film, and the cathode effect of the small second phase was not obvious under its influence. The Mg matrix dissolved uniformly and formed a more uniform and dense film. In addition, part of the film was porous and dissolved, and the chloride ion could pass through the film along with the defects. Therefore, for the uneven corrosion product film of the forged WE43 alloy, chloride ions were easier to penetrate and acted on the Mg substrate under the film.

## 5. Conclusions

(1) The extruded and forged WE43 have the same phase composition but different microstructures. The extruded WE43 alloy undergoes complete dynamic recrystallization, showing a uniform phase distribution. Due to the nonuniform segregation of rare earth elements, the forged WE43 alloy exhibits coarse grains and a larger phase size relative to the extruded WE43 alloy.
(2) Different cathode sizes might lead to different corrosion behaviors, and the large second phase has higher electrochemical activity than the pearl-shaped second phase. A strong microcouple is formed between the large second phase and the Mg matrix, which causes severe local corrosion.
(3) The surface film of the forged WE43 alloy fails in shorter time, while the surface film of the extruded WE43 alloy still maintains a good protective ability after a longer immersion time.
(4) The extruded WE43 alloy exhibits better corrosion resistance than the forged WE43 alloy, which is attributed to the synergistic effect of the microgalvanic corrosion behavior and the formed surface film.

## Figures and Tables

**Table 1:** The chemical composition of two deformed magnesium alloys.

State	Particle	Mg (at%)	Y (at%)	Nd (at%)	Gd (at%)	Zr (at%)
Extruded	I	86.47	4.78	8.13	0.37	0.25
	II	87.57	5.55	6.34	0.35	0.19
	III	95.84	1.93	1.33	0.41	0.49
	α-Mg	98.05	1.14	0.21	0.22	0.39
Forged	I	87.22	5.77	5.9	0.67	0.44
	II	86.51	6.45	5.87	0.84	0.33
	III	91.86	2.45	4.76	0.35	0.58
	α-Mg	98.20	1.17	0.26	0.20	0.17

**Table 2:** Fitting results of EIS spectra.

Status	R_s_ Ω cm^2^	Q_dl_ (μF cm^−2^ s^n−1^)	n_dl_	R_t_ Ω cm^2^	Q_f_ (μF cm^−2^ s^n−1^)	n_f_	R_f_ Ω cm^2^	R_L_ Ω cm^2^	L H cm^−2^	χ^2^
Extruded	11.81	1.71 × 10^−3^	0.70	909.6	1.40 × 10^−5^	0.93	1247	5051	1.16 × 10^4^	1.83 × 10^−4^
Forged	11.28	1.63 × 10^−5^	0.92	682.7	2.45 × 10^−3^	0.66	549	1225	8.21 × 10^4^	2.76 × 10^−4^

**Table 3:** The fitting result of the cathode polarization curve.

State	E_corr_ (V_SCE_)	I_corr_ (μA cm^−2^)	B_c_ (mV decade^−1^)
Forged	−1.82 ± 0.2	140.3 ± 5	319.8 ± 5
Extruded	−1.75 ± 0.1	33.1 ± 2	265.9 ± 3

## Data Availability

All data used to support the findings of this study are included within the article.
